# Two Optimized Methods for Efficient, Stable and Transient Transformation of Broccoli (*Brassica oleracea* Var. Italica)

**DOI:** 10.3390/plants15060978

**Published:** 2026-03-22

**Authors:** Alberto Coronado-Martín, Alejandro Atarés, Rosa Porcel, Lynne Yenush, José M. Mulet

**Affiliations:** Instituto de Biología Molecular y Celular de Plantas (IBMCP), Universitat Politècnica de València-Consejo Superior de Investigaciones Científicas, 46022 Valencia, Spainroporrol@upv.es (R.P.); lynne@ibmcp.upv.es (L.Y.)

**Keywords:** Broccoli, GFP, transformation, electroporation, protoplast, transgenic

## Abstract

Broccoli (*Brassica oleracea* var. italica) is an important crop valued for its nutritional and health-promoting properties, yet its biotechnological improvement is limited by low effectivity and genotype-dependent transformation protocols. The absence of reliable transient expression systems further constrains functional genomics and genome-editing applications. Here, we optimized regeneration and transformation protocols for different broccoli genotypes. Endoreduplication patterns in young tissues were analyzed by flow cytometry to identify suitable explants, and combinations of plant growth regulators were tested to develop an efficient organogenic medium. Stable transformation was achieved via *Agrobacterium tumefaciens* using nptII and eGFP markers. Cotyledons and hypocotyls up to day 7 showed similar endoreduplication patterns, with abundant 2n cells, but hypocotyls exhibited higher regeneration capacity. The optimized medium supported efficient organogenesis while maintaining diploidy. Transformation efficiency reached 10.4% in ‘S1’ and 2.8% in ‘Naxos’, highlighting genotype dependence. In parallel, a transient expression system was established using cotyledon-derived protoplasts and electroporation-mediated DNA delivery. GFP expression was confirmed through fluorescence microscopy, confocal imaging, and Western blotting. These protocols provide a robust toolkit for broccoli genetic manipulation, facilitating molecular biology studies in the native plant, functional genomics and genome-editing strategies, including CRISPR-based approaches.

## 1. Introduction

Broccoli (*Brassica oleracea* var. italica) is widely consumed for its nutritional and health-promoting phytochemicals, including glucosinolates, polyphenols, flavonoids, vitamins, minerals, and fiber. Over the past decade, global consumption has increased markedly, driven by growing consumer awareness of its health benefits and low caloric content. Worldwide, China, India, and the United States are the largest producers, with Spain being the leading producer in Europe [1]. In 2022, global production of broccoli and cauliflower exceeded 26 million tons [2]. Broccoli is cultivated in areas prone to aridification or salinization due to climate change. For instance, in Spain, it is grown along the southeastern Mediterranean coast [3]. This has led to intense research on the mechanism by which broccoli adapts to drought and salt stress [4,5]. It is also vulnerable to several fungal diseases, such as Alternaria leaf spot, clubroot, and downy mildew, as well as to Sclerotinia stem rot (also known as White mold), and to bacterial diseases, including black rot [6]. Therefore, to maintain or increase current broccoli production, biotechnological breeding is required, and CRISPR is likely to be an essential tool to achieve this objective [7]. Several descriptions of protocols for broccoli transformation are found in the literature as summarized in the review Kumar and Srivastava 2016 [8]. However, most of them exhibit very low efficiency or the protocol is genotype dependent. This low efficiency of transformation is particularly evident during the regeneration stage, where explants frequently release secondary metabolites into the culture medium, leading to polyphenolic oxidation, medium browning, and progressive explant necrosis. In addition, as mentioned above, the transformation step is also affected, typically resulting in very low transformation efficiencies. Nowadays, there are no GMO or CRISPR-edited broccoli varieties on the market, although a few descriptions have appeared in the literature [9,10], among others. However, there is no standard method for broccoli transformation, as each description in the literature employs a different strategy [11].

In this study, we have developed and improved an Agrobacterium-mediated medium for stable transformation, an electroporation method as an alternative to PEG-mediated transient protoplast transformation, and evaluated its efficiency across various genetic backgrounds. The presented stable transformation method improves existing stable transgenic plant development methodologies for gene overexpression studies or genome editing using CRISPR/Cas systems, representing the long-term goal of our work. Transient expression in broccoli protoplasts by electroporation provides a rapid system for studying broccoli proteins in their native state, particularly for subcellular localization analyses. This approach is analogous to the transient expression systems widely used in model species such as *Arabidopsis thaliana* or *Nicotiana benthamiana*, but it avoids potential artifacts that may arise when studying broccoli-specific genes in heterologous model organisms. We have used nptII as a selectable marker in broccoli tissues, and GFP as a reliable reporter for both transient and stable expression. Collectively, these advances provide essential tools for future genetic manipulation and functional genomics studies in broccoli, laying the groundwork for efficient biotechnological breeding of this economically important crop.

## 2. Results

### 2.1. Young Tissue Displays a Distinctly Lower Endoreduplication Profile

**Figure 1:**
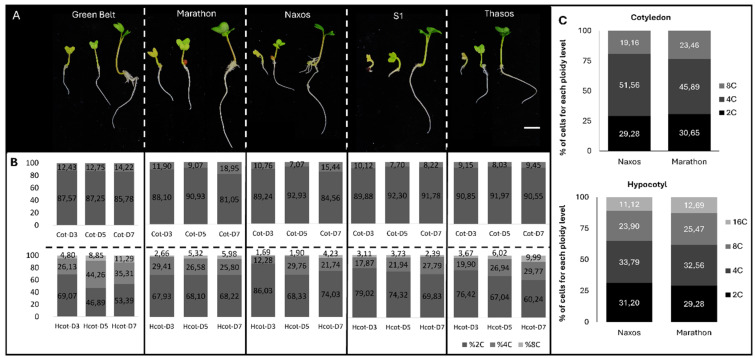
Polysomatic pattern analysis in cotyledons (Cot) and hypocotyls (Hcot) of five broccoli varieties (‘Green Belt’, ‘Marathon’, ‘Naxos’, ‘S1’, and ‘Thasos’) during the early stages of germination. (**A**) Seedlings at 3, 5, and 7 days after germination (D3, D5, and D7), when nuclear DNA content analyses were performed. Scale bar 1 cm. (**B**) Percentage distribution of nuclei with 2C, 4C, and 8C DNA contents, represented in grayscale. (**C**) Polysomy pattern of 10-day-old seedlings.

Selecting the appropriate explant type and developmental stage is crucial for minimizing endoreduplication and preventing the regeneration of plants with altered ploidy compared to the starting material. The nuclear DNA content of cotyledons and hypocotyls from five broccoli cultivars (‘Green Belt’, ‘Marathon’, ‘Naxos’, ‘S1′, and ‘Thasos’) was analyzed in a Partec CyFlow cytometer during early seedling development for three consecutive days (3.5, and 7) (Figure 1). Seedlings exhibited clear growth progression, characterized by rapid cotyledon expansion, radicle development, and a progressive color transition from pale yellow to green. All genotypes followed the same general pattern with minor differences in vigor (Figure 1A). Cotyledons for all five genotypes studied mainly exhibited 2C nuclei with a small percentage of 4C with limited changes over the first week (Figure 1B upper panel). In contrast, hypocotyls apart from having 2C and 4C nuclei, they also presented a proportion of 8C nuclei that gradually increased over time, reflecting active endoreduplication (Figure 1B lower panel). After 10 days, this endoreduplication was even more accurate with cotyledons also displaying 8C nuclei and hypocotyls reaching 16C, indicating tissue-specific increases in DNA content (Figure 1C). Altogether, these results underscore the importance of utilizing the youngest available tissue to minimize the effects of endoreduplication on regeneration.

### 2.2. Regeneration Responses in Broccoli Reveal Strong Genotype and Explant-Dependent Effects

To determine how genotype and explant type affect organogenesis in broccoli, an initial trial with three genotypes (‘Marathon’, ‘Naxos’, and ‘Thasos’) was performed using the eight regeneration media tested, containing each one different concentrations and combinations of plant growth regulators (PGRs) (Table 1). Hypocotyl explants consistently outperformed cotyledons in shoot regeneration across all genotypes (Supplementary Figure S2A,B). Thasos exhibited the highest mean regeneration values, whereas Marathon and Naxos exhibited lower responses. Several media induced excessive callus proliferation and widespread hyperhydricity, along with excessive polyphenolic oxidation, particularly in ‘Marathon’ and ‘Naxos’, which hampered shoot formation (Supplementary Figure S2B). Representative explants exhibited translucent, swollen tissues characteristic of hyperhydricity, indicating physiological stress under specific hormonal conditions. Significant effects of genotype, explant type, and their interaction on regeneration were observed, while the culture medium had no significant effect on the outcomes. Three-way ANOVA analysis and post hoc Tukey HSD comparisons are provided in Supplementary Table S1 and Table S2, respectively.

Some genotypes exhibited better regeneration, indicating that each protocol must be adapted for the specific genotype–explant combination. To avoid genotype-specific responses we tested NB1020 medium for adventitious regeneration as this medium has proven to be effective in our group for other plant species. Both hypocotyl and cotyledon explants remained healthier and less vitrified but produced abundant adventitious roots instead of shoots, indicating that this formulation supports rhizogenesis but not organogenesis (Supplementary Figure S2C).

### 2.3. Early Polysomatic Patterns Correlate with the Ploidy Level of Plants That Are Efficiently Regenerated

**Figure 2:**
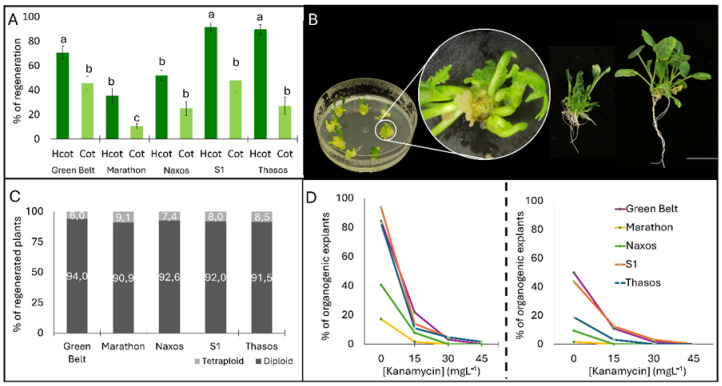
Regeneration capacity and ploidy analysis in five broccoli varieties. (**A**) Regeneration efficiency of hypocotyl (HC) and cotyledon (COT) explants from ‘Green Belt’, ‘Marathon’, ‘Naxos’, ‘S1’, and ‘Thasos’ cultured on NB0.02/20 organogenic medium. Bars represent mean regeneration percentage ± SE after 30 days in culture. Different lowercase letters indicate significant differences between treatments (Duncan test, *p* ≤ 0.05). (**B**) Representative stages of shoot regeneration, elongation, and rooting from organogenic explants. Scale bar: 1 cm. (**C**) Flow cytometry-based ploidy analysis of regenerated plants from each genotype. (**D**) Empirical determination of kanamycin concentration required to suppress shoot regeneration in hypocotyl (**left**) and cotyledon (**right**) explants for each genotype.

To reduce rhizogenesis and promote shoot organogenesis, a new regeneration assay was conducted using hypocotyl and cotyledon explants from the five initial broccoli cultivars, cultured on NB0.02/20 medium where the 1-naphthaleneacetic acid (NAA) concentration was reduced 50 times from NB1020 (Figure 2). Hypocotyl explants displayed significantly higher regeneration rates than cotyledons across all genotypes (Figure 2A). ‘S1’ and ‘Thasos’ were the most responsive, whereas ‘Marathon’ regenerated less efficiently. Shoot elongation and rooting proceeded successfully without detectable hyperhydricity, indicating that NB0.02/20 medium provides balanced hormonal conditions for healthy morphogenesis (Figure 2B). Flow cytometry of regenerated plants revealed predominantly diploid profiles (>90%), although spontaneous tetraploids occurred at low frequency (6–9%) (Figure 2C). Kanamycin-sensitivity assays showed that 15 mg L^−1^ was sufficient to partially inhibit the regeneration of most genotypes, suggesting that this sub-lethal concentration is best for transformation assays using nptII as a selection gene (Figure 2D). Regenerated shoots were efficiently multiplied on MB3 PGR-free medium (Supplementary Figure S3). Plants were propagated through both shoot apical meristems (SAM) and axillary buds and produced vigorous shoots that rooted readily on the same medium. Plants acclimatized successfully in greenhouse conditions, generating morphologically normal individuals.

### 2.4. Agrobacterium-Mediated Transformation and Verification of GFP Expression

**Figure 3:**
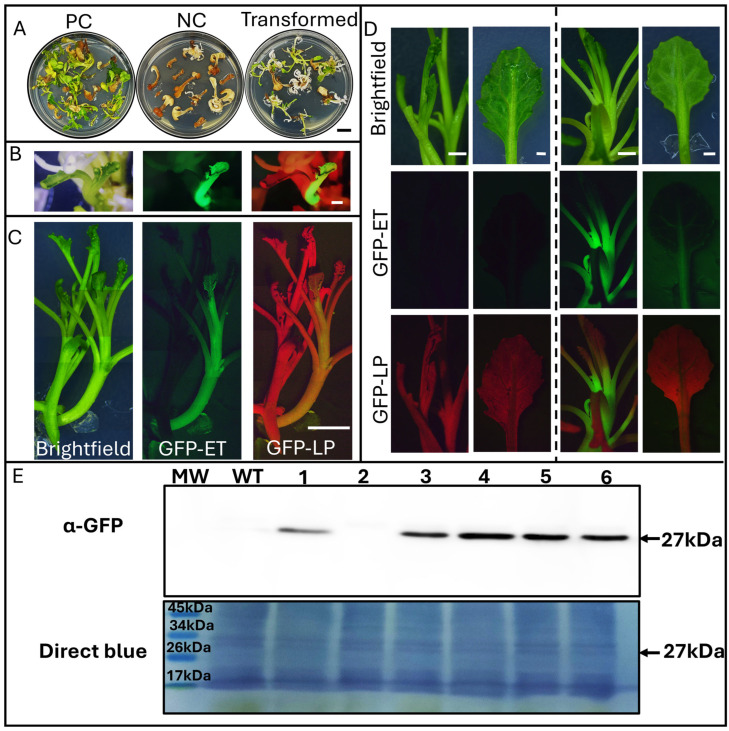
Agrobacterium-mediated transformation and regeneration of “S1” genotype broccoli explants. (**A**) Representative plates showing the positive control (PC, non-selective medium), the negative control (NC, 15 mg L^−1^ kanamycin), and explants inoculated with Agrobacterium carrying the plasmid under selective conditions (Transformed). Scale bar: 1 cm (**B**) Regenerating chimeric shoot observed under bright field (Brightfield), GFP emission (GFP-ET), and GFP long-pass (GFP-LP) filters. Scale bar: 1 mm. (**C**) Fully fluorescent transgenic shoot next to a wild type (WT) regenerant. Scale bar: 5 mm. (**D**) Macroscopic visualization of GFP fluorescence in the stem and leaves of WT and transgenic plants under the same filter sets. Scale bar: 1 mm. (**E**) Immunodetection of GFP protein in ‘S1’ transgenic broccoli lines. Western blot analysis of six putative transgenic events (TRG1–TRG6) using an anti-GFP antibody. Wild-type (WT) plants served as a negative control. Lower panels show total protein loading, and the molecular weight of the markers is indicated.

Transformation experiments confirmed the efficiency of the selection system as well as the reporter gene signal. Explants exposed to Agrobacterium produced both green (putative transgenic) and white (escape) shoots (Figure 3A). GFP fluorescence in regenerating tissues validated transgene expression, showing both chimeric shoots (Figure 3B) and solid transformants (Figure 3C), as well as in elongated plants (Figure 3D). We obtained a regeneration efficiency (number of explants with at least one fluorescent regeneration event) of 10.4% in the ‘S1’ genotype and 2.8% in ‘Naxos’. At the same time, no transformants were detected in any of the other three genotypes. These results strongly support the idea that, regardless of the high efficiency in some varieties, transformation is genotype-dependent. Western blot analysis further corroborated eGFP expression in five of six independent transformation events in ‘S1’, establishing the robustness of the transformation procedure (Figure 3E).

**Figure 4:**
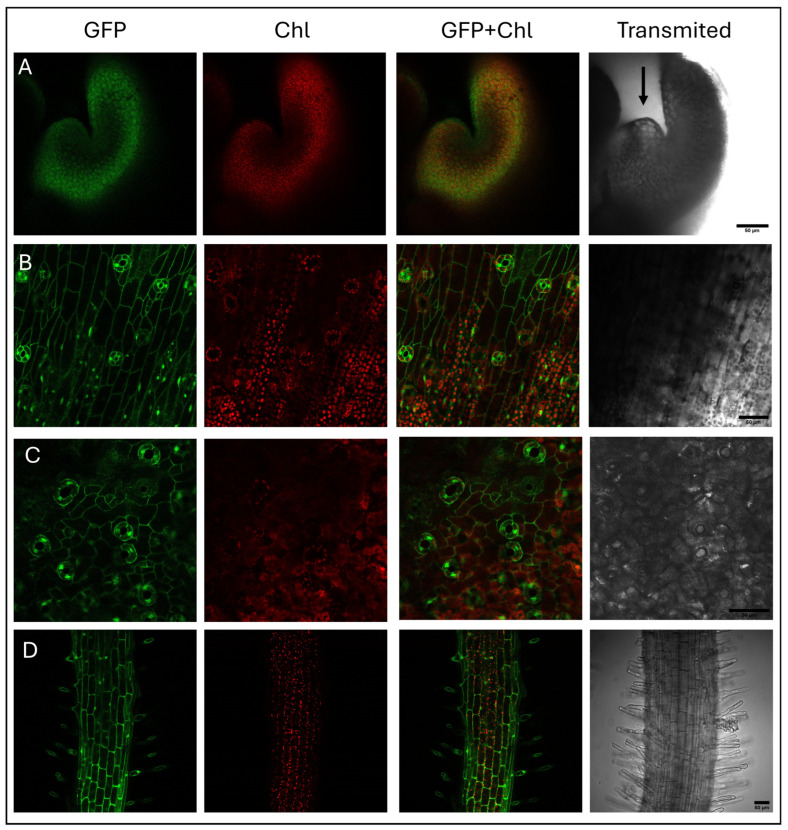
Confocal microscopy analysis of GFP expression in transgenic “S1” genotype broccoli tissues. GFP fluorescence (GFP; green) and chlorophyll autofluorescence (Chl; red) were examined in (**A**) Leaf primordia and the apical meristem (marked with an arrow) (**B**) leaf vein epidermal cells, (**C**) leaf epidermis cells, and (**D**) epidermic cells of a root segment. Merged GFP + Chl images show clear GFP expression across different cell types. GFP was detected with a laser capable of exciting this protein at 488 nm and allowing for its detection between 500 and 520 nm. Chlorophyll autofluorescence was also detected at 670–685 nm. Scale bar: 50 μm.

GFP presence was confirmed by confocal imaging (Figure 4). Different tissues were examined, including the apical meristem, vascular tissue, epidermis, and roots, which is consistent with vigorous p35S promoter activity.

### 2.5. Electroporation Enables Transient Transformation as an Alternative to PEG-Mediated Methods in Broccoli Protoplasts

**Figure 5:**
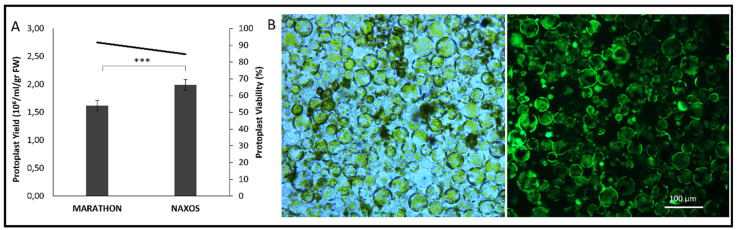
Isolation and viability of broccoli protoplasts. (**A**) Comparison of protoplast yield obtained from cotyledon tissue of two *Brassica oleracea* var. italica genotypes, ‘Marathon’ and ‘Naxos’. Data are expressed as the number of protoplasts (×10^6^/mL/g FW). The line represents the percentage of viable protoplasts. Asterisks indicate statistically significant differences (*** *p* < 0.001). (**B**) Representative images of freshly isolated broccoli protoplasts visualized under bright field (**left**) and using GFP filters (**right**) after FDA staining. Scale bar: 100 µm.

Once a robust method for stable transformation was developed, we attempted to establish a technique enabling transient transformation. As a prerequisite for creating a transient expression system suitable for localization and gene-function studies, we set out to isolate high-quality protoplasts from broccoli (Figure 5). Mesophyll protoplasts from cotyledons of ‘Marathon’ and ‘Naxos’ were successfully isolated yielding 1.5–2.0 × 10^6^ cells/mL/g FW with ‘Naxos’ producing significantly higher yields than ‘Marathon’ (Figure 5A). FDA staining confirmed high (>90%) viability and good structural integrity of the isolated protoplasts (Figure 5B).

**Figure 6:**
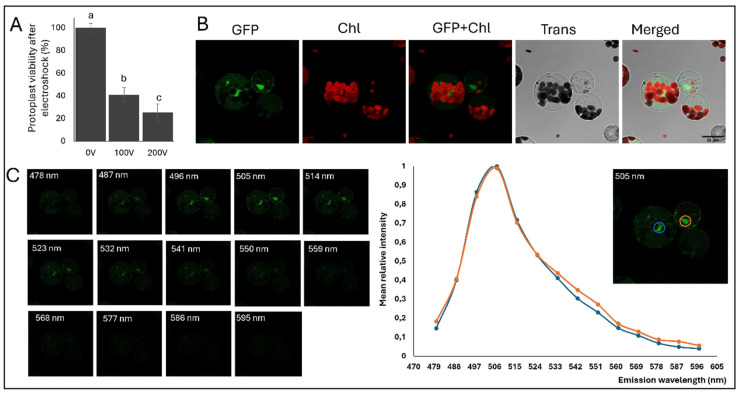
Optimization of transient transformation in *Brassica oleracea* protoplasts by electroporation. (**A**) Determination of optimal electroporation voltage based on protoplast viability after electric shock. Bars represent mean ± SE; different letters indicate statistically significant differences (*p* < 0.05). (**B**) Confocal microscopy of *B. oleracea* protoplasts transiently expressing the Pk2GW7–eGFP plasmid under optimized electroporation conditions. GFP signal in green and Chl signal in red. (**C**) Emission spectrum of two transformed protoplasts. The spectrum displays a characteristic GFP emission peak ~509 nm.

Attempts to transform broccoli protoplasts using PEG-mediated chemoporation were unsuccessful; therefore, we explored electroporation as an alternative method (Figure 6). To establish suitable parameters, we first assessed protoplast viability across different voltages and determined that 100 V yielded approximately 50% viability, representing a balance between survival and transformation efficiency (Figure 6A). Fluorescence was detected in both the cytosol and the nucleus (Figure 6B). The fluorescence emission spectrum (λ) obtained from transformed cells confirmed the presence of GFP-specific emission, validating successful reporter expression (Figure 6C).

### 2.6. Autofluorescent Structures Complicate the Interpretation of GFP-Like Signals in Broccoli Protoplasts

**Figure 7:**
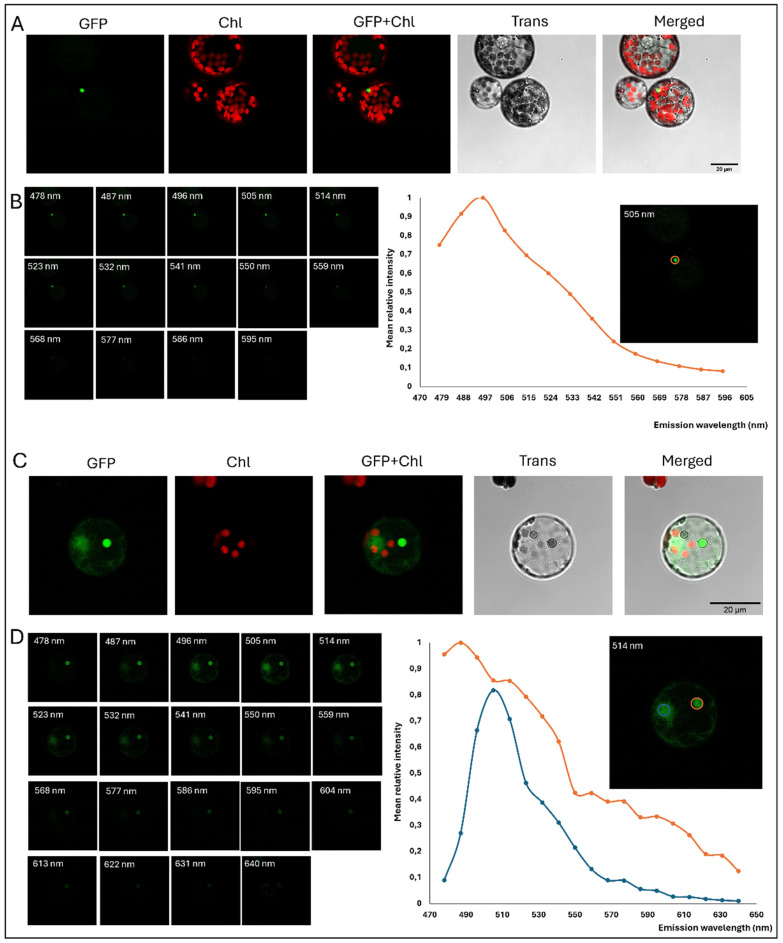
Autofluorescent structures in both non-transformed and transformed broccoli protoplasts. (**A**) Confocal images of broccoli untransformed protoplast showing an autofluorescent corpuscle under GFP channel. Scale bar: 20 µm. (**B**) Emission spectrum of the autofluorescent corpuscle with its peak at ~496 nm. (**C**) Confocal images of a transformed broccoli protoplast showing both transgenic fluorescence and an autofluorescent corpuscle under GFP channel. Scale bar: 20 µm. (**D**) Emission spectrum of the GFP signal (blue line) and the autofluorescent corpuscle signal (orange line). Scale bar: 20 µm.

When performing the experiments included in the present report, we found that confocal imaging revealed small autofluorescent corpuscles in non-transformed broccoli protoplasts that emitted within the GFP detection range (Figure 7). These corpuscles in non-transformed protoplasts (Figure 7A) displayed similar fluorescence as GFP in transformed broccoli (Figure 7C). However, the observed corpuscles did not have a marked nuclear morphology, they were clearly seen in any of both non-transgenic and transgenic protoplasts, had extremely defined boundaries and the nucleolus could not be seen inside these corpuscles. In addition, lambda scans confirmed the emission pattern of these corpuscles was different to the GFP signal (Figure 7B,D). This strongly indicates that such fluorescence does not result from genuine GFP expression and it is probably due to some secondary metabolite that is not present in plants such as Arabidopsis or Nicotiana, where it has not been reported before.

## 3. Discussion

The *Brassica oleracea* genome was first published in 2024 [12]. The possibility of genetic transformation, combined with the complete characterization of the genome, facilitates the use of molecular and new breeding techniques to improve this vital crop. Preliminary genome annotation indicated a high number of orphan genes [12], so there is great interest in developing methods to study broccoli genes in broccoli rather than in model systems such as Arabidopsis or Nicotiana. Another important aspect is the significant wild genetic diversity of *Brassica oleracea*, which constitutes a valuable genetic pool that can be utilized as a source of functional genes for introgression or the design of new GMO crops [11]. To date, several methods for transforming broccoli have been described in the literature. The biolistic transformation was first described in 1999 [13]. Still, there are no recent references to its use in the literature, probably because expensive equipment is required. Another problem with the biolistic technique is that it has been reported to have some side effects [14], and plant regeneration can represent a serious bottleneck [15].

There are also reports on the use of *Agrobacterium rhizogenes* to produce transgenic hairy roots from inoculated hypocotyl or stem explants [16]. This method can be used to develop stable transformed lines or for transient expression [17].

Transformation using Agrobacterium on explants is the most versatile technique, as it is easy to implement and cost-effective. Nevertheless, reported transformation efficiencies for broccoli using previously published Agrobacterium-mediated protocols are generally low and highly genotype-dependent. Thus, many parameters should be adjusted, such as the explant source (hypocotyls, cotyledons [18], peduncles [19], shoot tips [20]), the different culture media, and the marker for selection [21,22]. We found that one of the most limiting steps was the regeneration media, and formulated a new one. With this novel culture medium, we have been able to regenerate commercial genotypes that have not been transformed previously. This constitutes a significant improvement from previously published protocols. In our study, we have analyzed ploidy levels to characterize the starting plant material and ensure that the explants used for regeneration contained a low level of endoreduplication, thereby increasing the likelihood of regenerating predominantly diploid (2n) plants. Despite these precautions, our flow cytometry analysis revealed that a small proportion of regenerated plants were tetraploid. Maintaining diploid regenerants is important to preserve the original genomic constitution and genetic stability of the species. Polyploidization events during tissue culture may alter plant morphology, growth, and fertility, potentially confounding the interpretation of phenotypic effects associated with transgene expression or genome editing. This study also provides the first report of stable GFP expression in regenerated broccoli plants, thereby filling a long-standing gap in the literature, as GFP-expressing broccoli plants had not been previously generated. Significantly, we successfully transformed agronomically relevant genotypes, including ‘S1’ and ‘Naxos’. Some of the previously described transformation protocols are performed on non-commercial cultivars. Our method, although genotype-dependent as we could not transform phenotypes such as “marathon”, has proven to be effective in different genotypes, suggesting that it could also be effective in other not-evaluated genotypes.

Several reports are available for protoplast isolation and PEG-mediated DNA uptake for transient expression, which enable subcellular localization studies [23,24]. In most plant systems, mesophyll-derived protoplasts are commonly used for transient expression assays because they can be obtained in high yield and remain physiologically active. Here, we report the first successful electroporation of broccoli protoplasts, providing a new technique for rapid gene expression assays and transient GFP fusion analyses in this crop. By investigating these protoplasts, we have identified a characteristic of broccoli protoplasts that could lead to artifacts in the interpretation of images obtained by fluorescent microscopy. However, the use of confocal microscopy combined with lambda-scan analysis allowed us to clearly distinguish GFP fluorescence from autofluorescence, even when both appeared to emit within a similar wavelength range. Therefore, our results demonstrate that GFP can be reliably detected in broccoli tissues, including green tissues, when appropriate experimental conditions and controls are applied. These advances could be highly relevant for the development of protoplast transfection methods using ribonucleoproteins, as they would enable the generation of edited plants without producing GMOs, thereby saving both time and resources.

Beyond transgenic applications, methodologies described are directly relevant for the development of CRISPR-based genome editing and New Genomic Techniques (NGT) in broccoli. Although stable and transient transformation experiments reported in this study rely on nptII-mediated selection and GFP expression, the optimized regeneration and transformation protocols constitute essential prerequisites for genome editing strategies. In particular, successful electroporation of broccoli protoplasts opens the possibility of new editing technologies, such as the delivery of CRISPR–Cas ribonucleoprotein complexes, enabling targeted genome editing without stable integration of foreign DNA. Such DNA-free editing approaches would allow the recovery of edited plants lacking selectable marker genes, thereby aligning with current regulatory frameworks for NGT crops. Future work will focus on combining the regeneration system described here with transient CRISPR delivery methods, including ribonucleoprotein-based protoplast transfection or marker-free transformation strategies, to facilitate the implementation of genome editing in elite broccoli cultivars.

**Figure 8:**
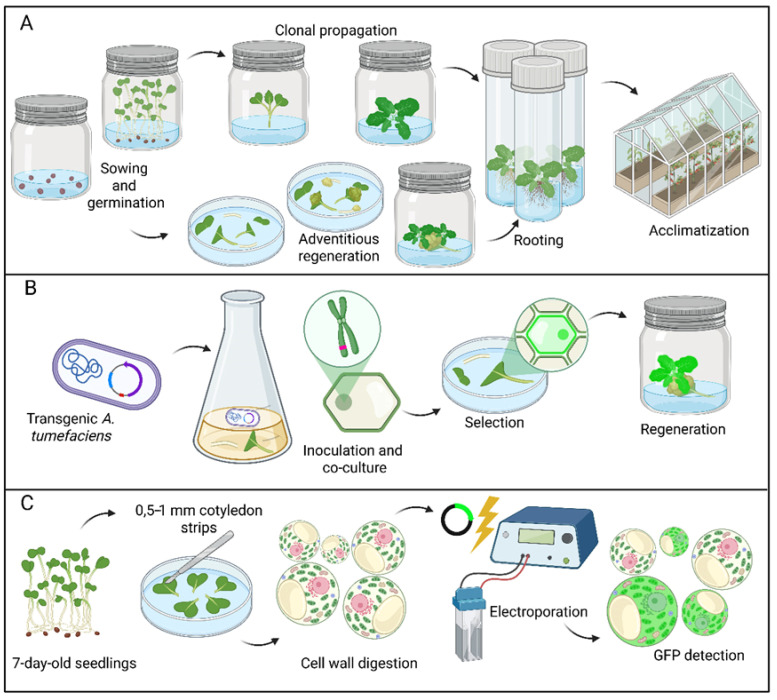
Workflow for the micropropagation and transformation in broccoli. (**A**) In vitro multiplication and acclimatization steps. (**B**) Stable genetic transformation workflow by the co-cultivation of explants with transgenic *Agrobacterium tumefaciens*. Red color represents the transgene. (**C**) Obtention and transient transformation of broccoli protoplasts from 7-day-old seedling cotyledons by electroporation. Created in BioRender. Green represents GFP signal. Red Chl signal.

To provide an overview of the experimental framework used in this work, we developed the workflow for *Brassica oleracea* var. italica (Figure 8). The scheme integrates and summarizes three complementary methodological approaches. We first established the basis for regenerating axenic cotyledon and hypocotyl explants from 7-day-old seedlings and then developed a method for acclimatizing the obtained propagules (Figure 8A). Once implemented, this protocol was used for stable transformation through co-cultivation with transgenic Agrobacterium tumefaciens, followed by the selection of transgenic events, shoot regeneration, rooting, and acclimatization (Figure 8B). In parallel, a transient expression pipeline using cotyledon-derived protoplasts was implemented by electroporation to enable rapid characterization of broccoli proteins attached to fluorescent reporters Figure 8C).

## 4. Materials and Methods

### 4.1. Plant Material and Culture Conditions

Seeds from five different broccoli genotypes (‘Green Belt’, ‘Marathon’, ’Naxos’, S1’, ‘Thasos’) were surface sterilized using 2.5% (*m*/*v*) sodium hypochlorite solution for 20–25 min with Tween 20. They were rinsed four times in sterile water and later sown on Ø 90 mm × h 90 mm rounded vessels containing MB3 medium (30 g·L^−1^ sucrose, 4.3 g·L^−1^ MS basal salts–Duchefa Biochemie, Haarlem, The Netherlands, 1 mg·L^−1^ THCl, 100 mg·L^−1^ myo-inositol, pH 5.7, 6.8 g·L^−1^ bacteriological agar). Seeds were incubated for germination at a light intensity of 70 μmol·m^−2^·s^−1^ under a 16 h light/8 h dark photoperiod at 25 ± 2 °C. These conditions were maintained throughout all experiments.

### 4.2. Ploidy Determination

Polysomatic patterns from 3-, 5-, and 7-day-old seedlings from all broccoli genotypes, as well as the ploidy level of regenerated shoots, were assessed by DNA quantification using a Partec CyFlow cytometer (Partec, Franklin Park, IL, USA). Cotyledons, hypocotyls, or young leaves were chopped with the addition of 0.2 mL nuclei isolation buffer (High Resolution DNA Kit, Solution A: Nuclei Isolation; Partec) and mixed with 0.8 mL staining buffer (High Resolution DNA Kit, Solution B: DAPI Staining; Partec). Before measuring, the suspension was filtered through a 50-µm nylon mesh (Nyblot).

### 4.3. Adventitious Regeneration

Hypocotyl and cotyledon explants from 7-day-old seedlings were cultured on MB3 basal medium containing different concentrations of plant growth regulators (Table 1). Each hypocotyl was cut into two individual explants around 1 cm each with the elimination of the apical meristem. Cotyledon explants consisted in petiole plus half of the cotyledon leaf, with a standard size of 1 cm long from the petiole to the middle of the cotyledon leaf. For each genotype and media, fifty explants were used, 25 from hypocotyl and 25 from cotyledon. Between 8 and 10 explants were cultured in each Petri dish. The regeneration media were designed to evaluate the effect of individual cytokinins (6-BA or ZR) and their interaction with auxin IAA. Therefore, treatments consisted of specific hormone formulations rather than all possible combinations of factor levels, so the experiment was not structured as a fully orthogonal factorial design. After testing these media, two additional ones that had been worked better for other species in the group were tested, NB1.0/2.0 and NB0.02/2.0, both containing the same previously described MB3 basal medium plus 1 mg·L^−1^ or 0.02 mg·L^−1^ 1-naphthaleneacetic acid (NAA) and 2 mg·L^−1^ 6-benzyladenine (BA), respectively. The organogenic response rate was assessed after 45 days. All experiments were performed in triplicate. To establish the minimal inhibitory concentration (MIC) for transgenic selection, same type hypocotyl and cotyledon explants were cultured on NB0.02/2.0 with increasing kanamycin concentrations (0, 15, 30, and 45 mg·L^−1^). Fifty explants were used per genotype and treatment tested distributed in 8–10 explants per Petri dish. MIC was evaluated by assessing the organogenic response after 45 days.

### 4.4. Rooting and Acclimatization

Adventitious shoots were individualized and rooted on Ø 90 mm × h 90 mm rounded vessels for proper growing into MB3basal medium free of PGRs. They were rinsed with deionized water to remove agar residues and transplanted into pre-moistened fertilized peat (Kekkilä Professional OPM 525W) combined with perlite (5:2). Plants were gradually acclimatized using a transparent plastic cup to greenhouse conditions: long-day photoperiod (16 h natural light supplemented with Osram Powerstar HQI-BT lamps, 400 W; Osram Licht AG, Munich, Germany), day/night temperatures of 24 °C and 18 °C.

### 4.5. Plasmids Used in This Study

We used the binary plasmid Pk2GW7–eGFP (Supplementary Figure S1), which includes the eGFP gene as a reporter [25], under the control of a strong and constitutive promoter (p35S from the cauliflower mosaic virus) and a selectable nptII marker enabling the selection of putatively transformed cells. This plasmid was used for both stable and transient transformation assays.

### 4.6. Genetic Transformation

Hypocotyl and cotyledon explants were inoculated with the disarmed Agrobacterium tumefaciens strain LBA 4404 harboring the Pk2GW7-eGFP plasmid on MB3 liquid medium supplemented with 3′5′-dimethoxy-4′hydroxyacetophenone at a final concentration of 200 μM (Sigma-Aldrich, St. Louis, MO, USA). They were then co-cultivated in MB3 liquid medium supplemented with acetosyringone at a final concentration of 200 μM in darkness for 48 h at 28 °C. They were then washed with MB3 liquid medium containing cefotaxime (500 mg·L^−1^). Selection was performed on NB0.02/2.0 medium supplemented with timentin (300 mg·L^−1^) and kanamycin (15 mg·L^−1^). Cultures were subcultured every two weeks onto the same medium until organogenic structures developed. Transformation efficiency was calculated as the ratio of explants exhibiting one or more transgenic events to the total number of explants. Organogenic shoots were transferred to Ø 90 mm × h 90 mm rounded vessels for proper growing into MB3 supplemented with timentin (300 mg·L^−1^) for three subcultures to ensure no Agrobacterium growth. Agrobacterium strain EHA105 was also assayed, but we were unable to regenerate plants from tissues infected with this strain, so we discarded this strain in subsequent experiments.

### 4.7. Protoplast Isolation and Electroporation

Mesophyll protoplasts were isolated from 7-day-old seedlings. Cotyledons were cut into 0.5–1 mm strips with a sharp razor blade and suspended in the digestion medium (DM) containing 0.5% cellulase Onozuka R-10, 0.1% pectolyase, 0.1% Bovine Serum Albumin, 20 mM KCl, 10 mM CaCl_2_, 20 mM MES, and 0.5 M mannitol, pH 5.7. They were then set to completely digest for four hours on an orbital shaker at 80 rpm in the dark at 24 °C. The same volume of W5 solution (154 mM NaCl, 125 mM CaCl_2_, 5 mM KCl, and 2 mM MES, pH 5.7) was added to stop the digestion. Protoplasts were passed through a 63 μm nylon mesh and then centrifuged at 2500 rpm for 3 min. The supernatant was discarded, and the pellet was resuspended in the same volume of W5. Protoplasts were washed twice and then resuspended in an electroporation medium (EM) volume sufficient to achieve a final concentration of 2 × 10^6^ cells/mL, as determined using a hemocytometer. EM medium contained 150 mM KCl, 4 mM CaCl_2_, 20 mM MES (pH 5.8), and 0.4 M mannitol. Protoplast viability was assessed with 5 µg·mL^−1^ Fluorescein diacetate (FDA). We determined the necessary voltage conditions to reduce viability to 50%, and 2 × 10^5^ protoplasts were electroporated with 20 μg of Pk2GW7–eGFP plasmid in a 2 mm electrode gap cuvette. A BTX electrocell manipulator 600 (Hawthorne, NY, USA) was used (100 V, 300 μF and 72 ohms, final pulse of τ = 22 ms). After electroporation, protoplasts were carefully resuspended in 1 mL W5 and cultivated on 3 cm diameter Petri dishes overnight. GFP presence was checked after 24 h.

### 4.8. GFP Expression and Molecular Analysis of Transgenic Plant

Fluorescence in regenerated transgenic plants and protoplasts was observed in macroscopic tissues using an MZ16F LEICA stereomicroscope (Wetzlar, Germany), and in different cell types and protoplasts by confocal Stellaris 8 FALCON (Leica), both equipped with a light source or laser capable of exciting this protein at 488 nm and allowing for its detection between 500 and 520 nm. Chlorophyll autofluorescence was also detected at 670–685 nm.

### 4.9. Western Blot Analysis

100 mg leaf samples from 6 independent transformation events and a wild-type of the ‘S1’ genotype were nitrogen-frozen and ground. Proteins were extracted using Laemmli buffer (2.5×). After boiling for 5 min at 95 °C, protein extracts were loaded into a 10% polyacrylamide gel for electrophoresis (SDS-PAGE). Proteins were then transferred to a nitrocellulose membrane. This membrane was incubated with the primary antibody (α-GFP Rabbit polyclonal PABG1, Chromotek, Planegg, Germany) at a final dilution of 1:10,000 in blocking buffer overnight. A secondary antibody (Anti-Rabbit IgG, peroxidase-linked NA934, Cytiva Life Sciences; Marlborough, MA, USA) at a final dilution of 1:10,000 and the ECL™ Prime Western Blotting were used to detect the immunocomplexes corresponding to the GFP-positive transformants.

### 4.10. Statistical Analysis

To evaluate the effects and potential interactions among explant type, medium, and genotype in the eight-culture media regeneration assay, a three-way ANOVA was performed, followed by Tukey’s HSD post hoc test. In the NB0.02/2.0 regeneration assay, a two-way ANOVA was used to assess the effects and interaction between explant type and genotype, followed by Tukey’s HSD test. Protoplast yield was analyzed using a Student’s *t*-test, while protoplast viability was evaluated through a one-way ANOVA with Tukey’s HSD for multiple-comparison analysis.

## 5. Conclusions

The most significant advances of this study are the optimization of a regeneration and transformation system that improves transformation efficiency in some broccoli genotypes, the first report of stable GFP expression as a reporter for broccoli stable transformation, and the development of an electroporation-based method for transient transformation of broccoli protoplasts as an alternative to PEG-mediated approaches. However, transformation efficiency remains strongly genotype-dependent, and we were unable to transform some cultivars, indicating that further work will be required to develop broadly applicable transformation systems for broccoli. Taken together, the methodological innovations presented here provide a versatile tool that will strengthen basic and applied research in broccoli improvement. These advances will significantly accelerate targeted genotype design and genome editing, while offering significant advantages for functional studies of genes of interest, including validation of candidates, subcellular localization through fusion proteins, and in vivo tracking of gene expression.

## Figures and Tables

**Table 1:** Composition of the different (1–8) basal MB3-derived media supplemented with varying concentrations of auxins (IAA) and/or cytokinins (6BA and ZR) used for adventitious organogenesis.

	IAA (mg L^−1^)	6BA (mg L^−1^)	ZR (mg L^−1^)
1	0	1	0
2	0.5	1	0
3	0	0	1
4	0.5	0	1
5	0	0	1.5
6	0.5	0	1.5
7	0	1.5	0
8	0.5	1.5	0

## Data Availability

The original contributions presented in this study are included in the article/Supplementary Material. Further inquiries can be directed to the corresponding author.

## Supplementary Materials

The following supporting information can be downloaded at: https://www.mdpi.com/article/10.3390/plants15060978/s1, Figure S1. Schematic representation of the binary plasmid Pk2GW7–eGFP used for both Agrobacterium-mediated and protoplast transformation of broccoli. Figure: S2. Regeneration response of three broccoli genotypes across explant types and culture media. Figure S3. Stages of micropropagation from regenerated broccoli shoots on MB3 medium. Table S1. Summary of the three-way ANOVA evaluating the effects of Genotype, Medium, and Explant, as well as their interactions, on the organogenic response. Table S2. Tukey HSD post hoc comparisons for Genotype and Explant factors.
