# Supplementary material for: Two Optimized Methods for Efficient, Stable and Transient Transformation of Broccoli (Brassica oleracea Var. Italica)
Source: Plants (Basel). 2026 Mar 22;15(6):978. doi: 10.3390/plants15060978 (PMC13030244; doi:10.3390/plants15060978)
Supplement: Supplementary file 1 [file plants-15-00978-s001.zip › plants-4189347-supplementary.pdf]

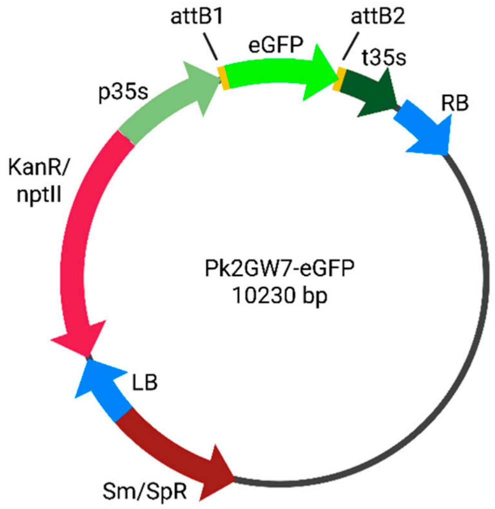

Supplementary Figure S1. Schematic representation of the binary plasmid Pk2GW7–eGFP used for both *Agrobacterium*-mediated and protoplast transformation of broccoli. Created in BioRender. Mulet, J. (2026) https://BioRender.com/

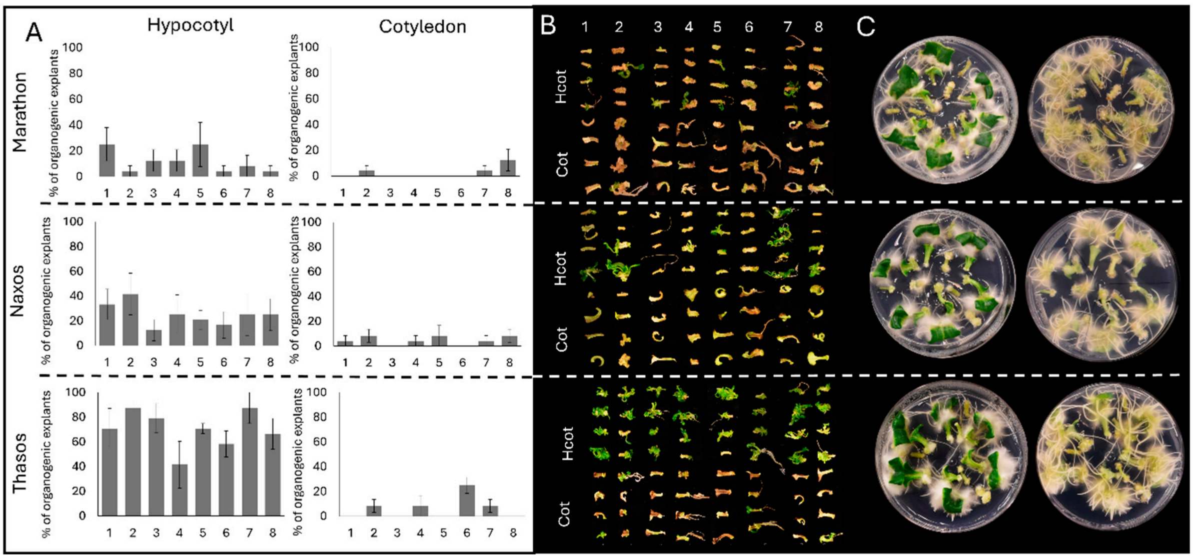

Supplementary Figure S2. Regeneration response of three broccoli genotypes across explant types and culture media. A) Regeneration efficiency of hypocotyl and cotyledon explants from the genotypes ‘*Marathon*’, ‘*Naxos*’, and ‘*Thasos*’ cultured on eight regeneration media. Bars represent mean regeneration percentage ± SE. **B)** Representative explants showing the morphological responses observed on each medium. **C)** One month old NB1020 medium regeneration of hypocotyl and cotyledon explants.

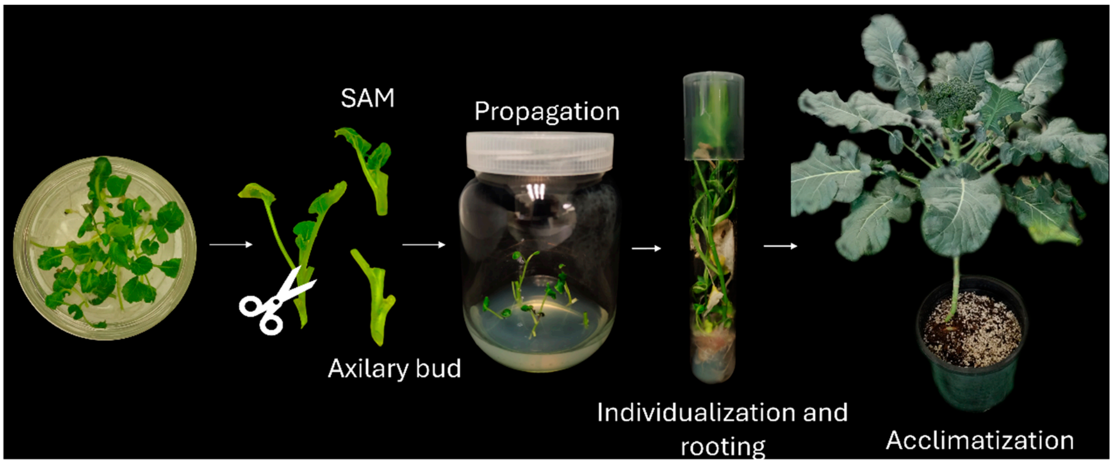

Supplementary Figure S3. Stages of micropropagation from regenerated broccoli shoots on MB3 medium. Sequential stages of shoot multiplication and plant recovery from regenerated broccoli explants. Axillary buds and shoot apical meristem (SAM) were transferred to MB3 medium for propagation. Newly developed shoots were rooted and acclimatized under greenhouse conditions.

Supplementary Table S1. Summary of the three-way ANOVA evaluating the effects of Genotype, Medium, and Explant, as well as their interactions, on the organogenic response. The table reports sum of squares, degrees of freedom, F-values, and associated p-values for each main factor and interaction.

**Factor**	**sum_sq**	**df**	**F**	**PR(>F)**
**C(Genotype)**	78,131944	2	51,421389	**2,59387E-19**
**C(Medium)**	4,777778	7	0,898407	0,5083509
**C(Explant)**	112,5	1	148,004393	**7,4554E-27**
**C(Genotype):C(Medium)**	10,534722	14	0,990467	0,466338
**C(Genotype):C(Explant)**	66,395833	2	43,697441	**6,55727E-17**
**C(Medium):C(Explant)**	7,055556	7	1,326717	0,2381915
**C(Genotype):C(Medium):C(Explant)**	11,318944	14	1,071023	0,3855621
**Residual**	182,333333	240		

Supplementary Table S2. Tukey HSD *post hoc* comparisons for Genotype and Explant factors. The table reports pairwise mean differences, adjusted p-values, confidence intervals, and significance decisions for all group contrasts following the three-way ANOVA.

**Tukey_Genotype**						
**group1**	**group2**	**meandiff**	**p-adj**	**lower**	**upper**	**reject**
Marathon	Naxos	0,2188	0,4035	-0,1816	0,6191	FALSE
Marathon	Thasos	1,1979	0	0,7976	1,5983	TRUE
Naxos	Thasos	0,9792	0	0,5788	1,3795	TRUE

**Tukey_Explant**						
**group1**	**group2**	**meandiff**	**p-adj**	**lower**	**upper**	**reject**
Cotyledon	Hypocot	1,25	0	0,9895	1,5105	TRUE
